# Prevalence of Smell or Taste Dysfunction Among Children With COVID-19 Infection: A Systematic Review and Meta-Analysis

**DOI:** 10.3389/fped.2021.686600

**Published:** 2021-08-03

**Authors:** Qingzi Yan, Dan Qiu, Xiang Liu, Xiaolan Guo, Yixiang Hu

**Affiliations:** ^1^Department of Pharmacy, Xiangtan Central Hospital, Xiangtan, China; ^2^Department of Social Medicine and Health Management, Xiangya School of Public Health, Central South University, Changsha, China

**Keywords:** children, smell dysfunction, taste dysfunction, COVID-19, prevalence

## Abstract

**Background:** Smell and taste dysfunctions are common and have been reported as an early indicator of COVID-19. The prevalence of smell and taste dysfunctions among children with COVID-19 varies greatly across studies, which remains to be summarized quantitatively. This review aimed at examining the pooled prevalence of smell or taste dysfunctions among children with COVID-19, summarizing possible causes of the inconsistencies in the current estimates.

**Methods:** Systematic searches of databases were conducted for literature published until 12 January 2021. Statistical analyses were performed using R software, the pooled prevalence was combined using random effects model. The Loney criteria were used for quality assessment.

**Results:** A total of 18 eligible studies were included. The results showed that the pooled prevalence of smell dysfunction among children with COVID-19 was 15.97% (95% CI: 8.18–23.77%), the pooled prevalence of taste dysfunction among children with COVID-19 was 9.20% (95% CI: 4.25–14.16%), the pooled prevalence of smell or taste dysfunction among children with COVID-19 was 15.50% (95% CI: 10.30–20.70%) and the pooled prevalence of smell and taste dysfunction among children with COVID-19 was 20.21% (95% CI: 14.14–26.28%). Higher smell or taste dysfunction rates were associated with being female, younger age, smaller sample size, patients in Asia, and with comorbidities.

**Conclusions:** Evidence suggests that smell or taste dysfunctions were common among children with COVID-19. Further research is needed to identify effective strategies for preventing and treating smell and taste dysfunctions among children with COVID-19.

## Introduction

Caused by severe acute respiratory syndrome coronavirus 2 (SARS-CoV-2), Coronavirus disease 2019 (COVID-19) was declared a global pandemic by the World Health Organization on March 11, 2020 (1). As of March 23, 2021, COVID-19 has infected over 122 million people worldwide, with over 2.7 million associated deaths (2). As cases of the novel coronavirus continue to rise, the way in which healthcare is practiced has been significantly impacted (3). Given that the ongoing COVID-19 pandemic rapidly progresses across the whole world, quickly obtaining accurate information on symptoms and their progression is important.

Clinicians and scientists from all over the world have been producing evidence to understand the epidemiology, clinical profile and prognostic factors of COVID-19. Based on early reports about clinical symptoms between January and February 2020, COVID-19 patients commonly presented with cough, fever, and fatigue. Upper respiratory tract symptoms such as rhinorrhea, sore throat and diarrhea were relatively uncommon (4). In late March 2020, there were anecdotal reports suggesting smell and taste dysfunctions as the early symptoms of COVID-19 patients (4, 5). Chemosensory functions, which are usually known as smell and taste functions, are the major pathways for mammals to sense and respond to chemical compounds in the environment, such as flavor, smell, and stimulant (6). COVID-19-related smell or taste dysfunctions have been described as a sudden onset, and may occur with or without other key symptoms (7). Due to increasing awareness of smell or taste dysfunctions as potential early symptoms of COVID-19 infection, “new loss of smell or taste” was added to its list of symptoms that may appear 2–14 days after exposure to COVID-19 (8). Understanding the prevalence of smell or taste dysfunction among COVID-19 patients is quite important, which may lead to increases in clinic visits and smell or taste testing due to concerns for COVID-19 (9).

Hoang et al. reported the pooled prevalence of smell and/or taste dysfunctions among adults with COVID-19 in their reviews (7, 10, 11), and the results were updated by von Bartheld et al., they indicated that the pooled prevalence of smell and/or taste dysfunctions among adults with COVID-19 ranged from 43.00 to 47.40% (12). For Children with COVID-19, however, the prevalence of smell or taste dysfunctions varies greatly across studies (13, 14). Additionally, the possible causes of the inconsistencies (such as age, gender, comorbidity, severity of the disease) in the current estimates among children with COVID-19 between different studies remained unclear. As the epidemic situation has been changing and the number of COVID-19 patients has been increasing, we think ongoing surveillance is essential. In order to provide more reliable prevention, it is necessary to determine a more accurate estimation of the prevalence of smell and/or taste dysfunction among children with COVID-19, and to explore the possible causes of the inconsistencies in the current estimates.

This review aimed at examining the pooled prevalence of smell and/or taste dysfunctions among COVID-19 patients aged <18 years, summarizing possible causes of the inconsistencies (such as age, gender, sample size, health status) in the current estimates, and to provide a reference for COVID-19 and possible outbreak of similar infectious diseases in the future.

## Materials and Methods

This review was prepared in accordance with the PRISMA guideline and Meta-analyses Of Observational Studies in Epidemiology (MOOSE) guidelines (15, 16). See Supplementary Tables 1, 2 for the PRISMA checklist and MOOSE checklist.

### Search Strategy

Electronic searches with PubMed, EMBASE, Web of Science, the Cochrane Library, Chinese National Knowledge Infrastructure (CNKI) and PsycArticle were independently conducted by two reviewers. The following search terms were used: “smell dysfunction” (including smell loss, smell disorder, olfactory dysfunction, etc.); “taste dysfunction” (including taste loss, taste disorder, gustatory dysfunction, etc.); “COVID-19” (including COVID-19, SARS-CoV-2, Coronavirus disease 2019 etc.); children (including child, newborns, teenager, adolescent, youngster, etc.). See Supplementary Data for a full search strategy. Restrictions on the publication date were set, only studies published between 1 December 2019 and 30 October 2020 were searched for. An update search was conducted on 12 January 2021. See Supplementary Materials for the details.

Given that this field is developing rapidly, the preprint servers medRxiv for studies published between Jan 1, 2020, and October 30, 2020, with the term “coronavirus” or “COVID-19” in the title or abstract were also searched for.

### Study Selection

Studies were included if they met the following criteria: (1) the study was an observational study; (2) information about prevalence of smell or taste dysfunction among children with COVID-19 was provided; (3) the full article was written in English or Chinese; (4) the participants were aged <18 years old. Studies were excluded: (1) if the report was a review, meta-analysis or protocol; (2) if the study with a case series that reported only selected cases having smell or taste dysfunction.

### Data Extraction

Two reviewers (DQ and QZY) checked the titles, abstracts and full-texts of the initial search results independently. Data were extracted on first author, year of publication, country or area, sample size, response rate, percentage of male participants, average age of participants, instruments used to identify smell or taste dysfunction, prevalence of smell and/or taste dysfunctions, percentage of smokers, percentage of patients with comorbidity, quality score of the included studies, etc. Any discrepancies that emerged in these procedures were discussed and resolved by involving a third reviewer (XL).

### Quality Assessment

Two independent reviewers (XLG and YXH) used the established guidelines, the Loney criteria, to evaluate the methodological quality of the included studies, which has been widely used to evaluate observational studies (17, 18). The included papers were scored according to eight criteria, such as study design, definition of participants, response rate, sampling method, sample size, appropriateness of measurement and analysis. The scores range from 0 to 8, with a score of 0–3 as low quality, 4–6 as moderate, and 7–8 as high (19). See Supplementary Table 4 for details on the quality assessment.

### Statistical Analyses

When data were available for three or more studies, prevalence was combined (20). When there were four or more studies, quantitative subgroup analysis was conducted (21). All the statistical analyses were performed using the “meta” (4.12-0) and “metafor” package (2.4-0) of R version 4.0.2. Between-study heterogeneity was evaluated by Cochran's Q test and quantified by the *I*^2^ statistic, with values 50% or more indicating possible moderate heterogeneity (22, 23). As we expected considerable heterogeneity, the pooled prevalence of smell and/or taste dysfunctions was combined using random effects model. If more than one dataset was reported for the same group of participants, the outcomes that were assessed at the baseline were used. In order to compare the prevalence from different studies (such as age, gender, sample size, health status, etc.), we conducted quantitative subgroup analysis. The difference between subgroups was examined using the Cochran's Q chi-square tests. When each group consists of only one study, quantitative subgroup analysis was not performed. Publication bias was investigated by funnel plot and Egger's test when there were 10 or more studies included. If publication bias was observed across studies, then the “trim and fill” method was applied (24). To evaluate the consistency of the results, sensitivity analysis was performed by removing each study individually. All the statistical tests were 2-sided, with a significance threshold of *P* < 0.05.

## Results

**Figure 1:**
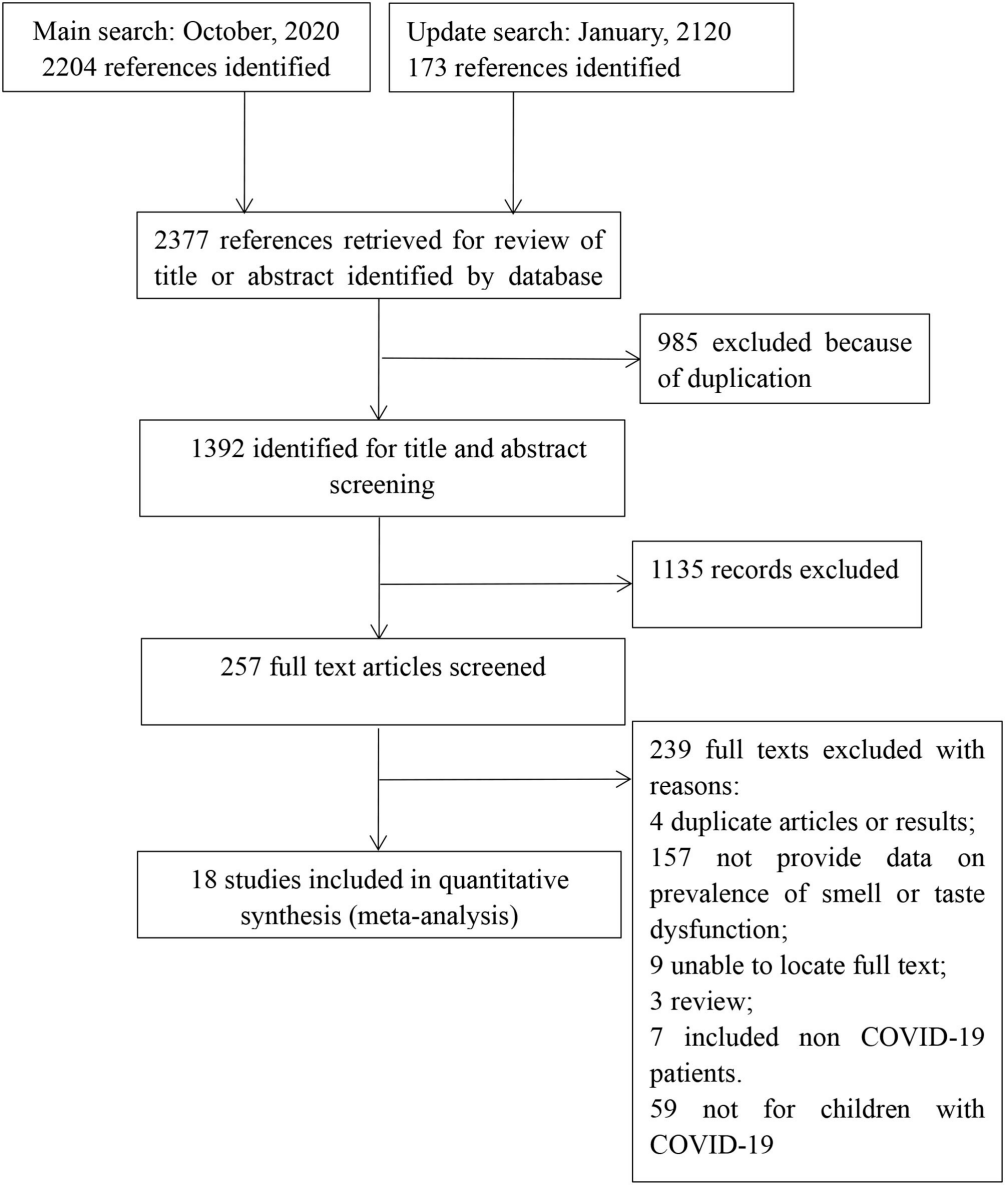
Flow of studies through review.

From the initially identified 2,377 records, 1,392 records remained after duplicates were excluded. One thousand one hundred thirty-five references were excluded on the basis of the title or abstract, leaving 257 full-text studies for further scrutiny. Of these, 18 met the selection criteria. Two hundred and thirty-nine studies were excluded due to the following reasons: no data on prevalence of smell and/or taste dysfunction (*n* = 157); duplicate publications (*n* = 4); no full-text (*n* = 9); review (*n* = 3); not for COVID-19 patients <18 years old (*n* = 59); included non-COVID-19 patients (*n* = 7). Finally, 18 articles were included for analysis. See Figure 1 for the details.

### Study Characteristics

As presented in (Table 1), 18 articles met the inclusion criteria. Of the included studies, 15 were cross sectional studies, two were longitudinal designs and one was case control studies. Most of the included studies were from Asia, such as China, South Korea, and Turkey. See Table 1 for the details. From the 18 papers, no study was rated as high quality, 16 (88.88%) were rated as moderate, and two (11.12%) were rated as low quality. Details of the methodological quality assessments of all 18 studies are shown in Supplementary Tables 3, 4.

**Table 1:** Study characteristics of included studies.

**References**	**Country/area**	**Study design**	**Effective sample**	**Study characteristics**	**Prevalence**	**Quality score**
Rusetsky et al. (25)	Russia	Cohort study	79	Response rate: 91.13% Percentage of male participants: 46.80% Mean age: 12.90 ± 3.40 Diagnosis assessment: the SNOT-22/Sniffin' Sticks” test	Smell dysfunction: 52/72 Taste dysfunction: 54/79	5
Qiu et al. (13)	China, Germany, France	Cross-sectional study	27	Response rate: / Percentage of male participants: / Mean age: 16.60 ± 0.70 Diagnosis assessment: self-report (questionnaire)	Smell dysfunction: 3/27 Taste dysfunction: 0/27 smell or taste dysfunction: 6/27 smell and taste dysfunction: 10/27	5
Li et al. (26)	China	Cross-sectional study	39	Response rate: / Percentage of male participants: 60% Mean age: 8.0 Diagnosis assessment: / Percentage of smokers: 44.0%	Smell or taste dysfunction: 2/39	5
Laws et al. (27)	America	Cross-sectional study	19	Response rate: / Percentage of male participants: 39% Mean age: 13 Diagnosis assessment:/ Percentage of smokers: 31.2% Percentage of mild or moderate patients: 31.57%	Smell dysfunction: 6/19 Taste dysfunction: 4/19	3
Kumar et al. (28)	India	Cross-sectional study	141	Response rate: / Percentage of male participants:58.9 Mean age: 15.2 Diagnosis assessment: self-report	Smell dysfunction: 34/141 Taste dysfunction: 34/141 smell or taste dysfunction: 40/141 smell and taste dysfunction: 28/141	4
Krajcar et al. (29)	Croatia	Cross-sectional study	151	Response rate: / Percentage of male participants: 33.1 Mean age: 10.0 Diagnosis assessment: self-report Percentage of mild or moderate patients: 88.07% Percentage of patients with comorbidity: 24.50%	Smell or taste dysfunction: 27/151	4
Korkmaz et al. (30)	Turkey	Cohort study	81	Response rate: / Percentage of male participants: 59.25 Mean age: 9.5 Diagnosis assessment: self-report Percentage of patients with comorbidity: 4.93%	Smell dysfunction: 1/81	4
King et al. (31)	Canada	Cross-sectional study	1,987	Response rate: 87.76 Percentage of male participants: 49.8 Mean age: 9.3 ± 5.2 Diagnosis assessment: self-report	Smell or taste dysfunction: 153/1,987	5
Kilani et al. (32)	Jordan	Cross-sectional study	61	Response rate: / Percentage of male participants: 60.6 Mean age: / Diagnosis assessment: self-report	Smell dysfunction: 9/61 Taste dysfunction: 5/61	4
He et al. (33)	China	Cross-sectional study	35	Response rate:100% Percentage of male participants: 66.67 Mean age: 7.1 ± 4.2 Diagnosis assessment: self-report	Smell or taste dysfunction: 1/35	4
Han et al. (34)	South Korea	Cross-sectional study	74	Response rate: / Percentage of male participants: 58 Mean age: 11 Diagnosis assessment: self-report Percentage of mild or moderate patients: 72.52%	Smell dysfunction: 4/74 Taste dysfunction: 8/74	4
				Percentage of patients with comorbidity: 8.10%		
Goss et al. (35)	America	Cross-sectional study	26	Response rate: 100% Percentage of male participants: 62.0 Mean age: 8 Diagnosis assessment: self-report Percentage of patients with comorbidity: 100%	Smell dysfunction: 2/26	4
Gaborieau et al. (36)	France	Cross-sectional study	157	Response rate: 93.65% Percentage of male participants: 59.9 Mean age: 0.5 Diagnosis assessment: self-report Percentage of patients with comorbidity: 28.02%	Smell or taste dysfunction: 7/157	4
Duarte-Salles et al. (14)	France, Germany, Spain, South Korea and the United States	Cross-sectional study	58,963	Response rate: / Percentage of male participants: / Mean age: / Diagnosis assessment:/	Smell or taste dysfunction: 267/58963	4
Concheiro-Guisan et al. (37)	Spain	Case-control study	33	Response rate:/ Percentage of male participants: 61 Mean age: 8.4 Diagnosis assessment: self-report	Smell dysfunction: 5/33 Taste dysfunction: 4/33	3
Chua et al. (38)	South Korea	Cross-sectional study	91	Response rate:/ Percentage of male participants: 58.2 Mean age: 10.8 ± 5.42 Diagnosis assessment: self-report	smell dysfunction: 4/91 taste dysfunction: 8/91	4
Chua et al. (38)	Hong Kong	Cross-sectional Study	88	Response rate: / Percentage of male participants: 58 Mean age: 12.9 ± 5.2 Diagnosis assessment: self-reported	Smell dysfunction: 7/88 taste dysfunction: 6/88	4
Arslan et al. (39)	Turkey	Cross-sectional study	176	Response rate: / Percentage of male participants: 55.7 Mean age: 6.5 Diagnosis assessment: self-report Percentage of mild or moderate patients: 65.9% Percentage of patients with comorbidity: 1.7%	Smell or Taste dysfunction: 15/176	4
Adedeji et al. (40)	Nigeria	Cross-sectional study	59	Response rate: / Percentage of male participants: 52.8 Mean age: 12.63 ± 4.31 Diagnosis assessment: self-report Percentage of mild or moderate patients: 39.63%	Smell dysfunction: 6/59 Taste dysfunction: 1/59	4

### Pooled Prevalence of Smell and/or Taste Dysfunctions

**Figure 2:**
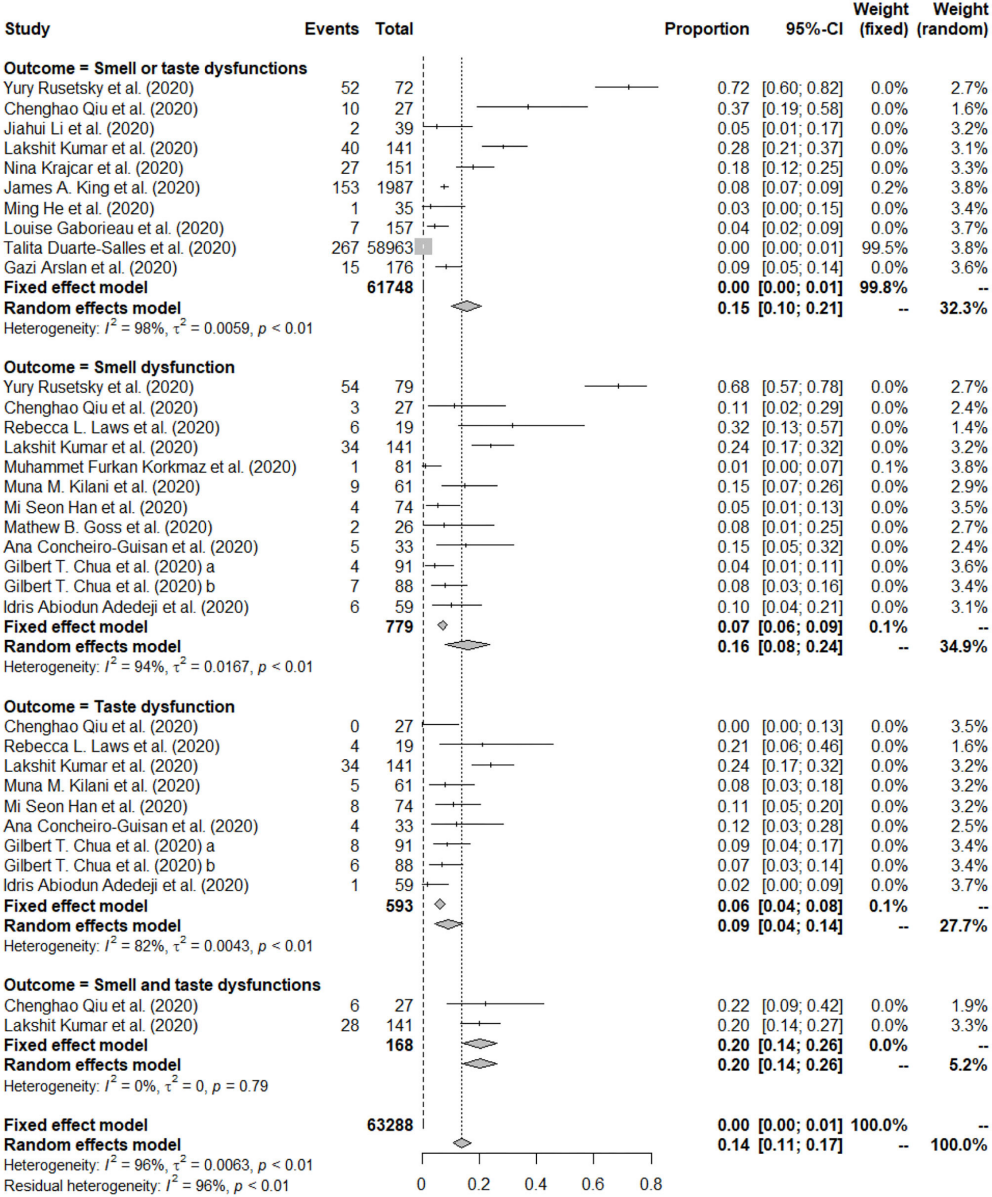
Forest plot of pooled prevalence of smell or/and taste dysfunction.

The forest plot in Figure 2 depicts the details. A total of 18 studies reported prevalence of smell and/or taste dysfunction, most of them reported more than one type of prevalence among children with COVID-19. Specifically, 11 of the included studies reported the prevalence on smell dysfunction (13, 25, 27, 28, 30, 34–36, 40), 9 of the included studies reported prevalence on taste dysfunction (13, 27, 28, 32, 34, 37, 38, 40), 10 of the included studies reported prevalence on smell or taste dysfunction (13, 14, 25, 26, 28, 29, 31, 33, 36, 39), and 2 of the included studies reported prevalence on smell and taste dysfunction (13, 28). Thus, four different types of prevalence were analyzed.

The prevalence of smell dysfunction reported among the included studies ranged from 1.23 to 68.35%. A total of 779 children with COVID-19 were identified in the 12 articles, of which 135 were reported with smell dysfunction. The random effects model was used to determine the pooled prevalence (*I*^2^ = 94.40%, *P* < 0.001), the pooled prevalence of smell dysfunction among children with COVID-19 was 15.97%, with a 95% CI of 8.18–23.77%. See Figure 2 for the details.

The prevalence of taste dysfunction reported among the included studies ranged from 0.00 to 24.11%. A total of 593 children with COVID-19 were identified in the 9 articles, of which 70 were reported with taste dysfunction. The random effects model was used to determine the pooled prevalence (*I*^2^ = 82.00%, *P* < 0.001), the pooled prevalence of taste dysfunction among children with COVID-19 was 9.20%, with a 95% CI of 4.25–14.16%. See Figure 2 for the details.

The prevalence of smell or taste dysfunction reported among the included studies ranged from 0.45 to 72.22%. A total of 61,748 children with COVID-19 were identified in the 10 articles, of which 574 were reported with smell or taste dysfunction. The random effects model was used to determine the pooled prevalence (*I*^2^ = 98.00%, *P* < 0.001), the pooled prevalence of smell or taste dysfunction among children with COVID-19 was 15.50%, with a 95% CI of 10.30–20.70%. When an extreme outlier study (with a prevalence <1%) was removed (14), the pooled prevalence of smell or taste dysfunction was 18.65% (95% CI: 11.21–21.60%), and the *I*^2^ statistic was 96.10%. See Figure 2 and Supplementary Figure 5 for the details.

The prevalence of smell and taste dysfunction reported among the included studies ranged from 19.86 to 22.22%. A total of 168 children with COVID-19 were identified in the 2 articles, of which 34 were reported with both smell and taste dysfunction. The random effects model was used to determine the pooled prevalence (*I*^2^ = 0.00%, *P* < 0.001), the pooled prevalence of smell and taste dysfunction among children with COVID-19 was 20.21%, with a 95% CI of 14.14–26.28%. See Figure 2 for the details.

### Subgroup Analysis for the Included Studies

Quantitative subgroup analyses were conducted for three outcomes, including smell dysfunction, taste dysfunction, and smell or taste dysfunction. The details of subgroup analyses were presented in Tables 2, 3 and Supplementary Table 5.

**Table 2:** Subgroup analysis for smell dysfunction.

**Subgroup**	**Number of studies**	**Pooled prevalence % (95% CI)**	***I*^**2**^ (%)**	**Test of difference within each subgroup**	
				***Q***	***p***
**Mean age**				3.35	0.067
0−10	3	6.34 (0.01–14.50)	89.60		
>10	8	19.64 (7.98–31.31)	88.30		
**Percentage of male participants (%)**				5.08	0.024
0–50	2	51.14 (15.17–87.11)	87.10		
>50	9	9.41 (4.68–14.14)	99.00		
**Area**				1.39	0.706
Asia	7	17.30 (6.51–28.09)	96.80		
America	2	17.80 (0.01-40.97)	75.30		
Europe	1	15.15 (2.92–27.38)	-		
Africa	1	10.87 (2.46–17.88)	-		
**Percentage of patients with mild or moderate COVID-19 (%)**				2.41	0.120
0–50	3	13.93 (4.47–23.38)	44.40		
>50	1	5.41 (0.03–10.56)	-		
**Percentage of patients with comorbidities (%)**				0.81	0.369
0–10	2	2.67 (0.01–6.56)	51.60		
>10	1	7.69 (0.01–17.93)	-		
**Quality score**				0.45	0.500
0–3	2	15.04 (6.62–23.45)	43.40		
4–6	10	21.09 (5.62–36.56)	95.20		

**Table 3:** Subgroup analysis for taste dysfunction.

**Subgroup**	**Number of studies**	**Pooled prevalence % (95% CI)**	***I*^**2**^ (%)**	**Test of difference within each subgroup**	
				***Q***	***p***
**Mean age**				2.91	0.088
0–10	2	4.82 (0.59–9.05)	0.00		
>10	5	11.19 (5.22–17.16)	64.70		
**Percentage of male participants (%)**				1.25	0.255
0–50	1	21.05 (2.72–39.38)	–		
>50	7	9.96 (4.47–15.44)	82.80		
**Area**				13.22	0.004
Asia	1	21.05 (2.72–39.38)	–		
America	5	11.55 (5.72–17.38)	76.30		
Europe	1	12.12 (0.99–23.26)	–		
Africa	1	1.69 (0.01–4.99)	–		
**Quality score**				1.27	0.259
0–3	2	14.53 (5.01–24.05)	0.00		
4–6	7	8.25 (2.90–13.20)	85.30		

There was no significant difference in the prevalence of smell dysfunction between different ages (6.34 vs. 19.64%; *Q* = 5.09, *P* > 0.05). Furthermore, there were significant differences in the pooled prevalence of smell dysfunction between different gender, female was associated with higher prevalence of smell dysfunction. Studies with a lower proportion of males reported a higher prevalence (51.14 vs. 9.41%; *Q* = 5.08, *P* = 0.024). Also, studies with a lower proportion ( ≤ 10%) of patients with comorbidities reported a lower prevalence of smell dysfunction, but the difference was not significant (2.67 vs. 7.69%; *Q* = 0.81, *P* = 0.369). Higher percentage of children with mild or moderate COVID-19 (>50%) was associated with lower prevalence of smell dysfunction, but the difference was not significant (13.93 vs. 5.41%; *Q* = 2.41, *P* = 0.120). There was no significant difference in the prevalence of smell dysfunction between different regions (*Q* = 1.39; *P* = 0.706). In addition, no significant difference in the prevalence of smell dysfunction between studies with different quality score was observed (15.04 vs. 21.49%; *Q* = 0.45, *P* = 0.500).

Older age was associated with higher prevalence of smell dysfunction, but the difference in the prevalence of taste dysfunction between different ages was not significant (4.81 vs. 11.19%; *Q* = 2.91; *P* = 0.088). There was no significant difference in the prevalence of taste dysfunction between different genders (21.05 vs. 9.96%; *Q* = 1.25, *P* = 0.255). Also, significant difference in the prevalence of taste dysfunction between different regions was observed, patients in Asia reported highest prevalence of taste dysfunction while patients in Africa reported lowest prevalence of taste dysfunction (21.05 vs. 11.55 vs. 12.12 vs. 1.69%; *Q* = 13.22, *P* = 0.004). In addition, no significant difference in the prevalence of taste dysfunction between studies with different quality score was observed (14.53 vs. 8.25%; *Q* = 1.27, *P* = 0.259).

For smell or taste dysfunction, age, sample size, and percentage of mild or moderate patients were found to be moderating variables for the prevalence, while gender, percentage of patients with comorbidities and area were not moderating variables for the prevalence; the details were presented in Supplementary Table 5.

### Publication Bias and Sensitivity Analysis

Funnel plots for different outcomes (smell dysfunction, and smell or taste dysfunction) were presented in Supplementary Figures 1, 2. The results of visual inspecting funnel plots and the Egger's test for smell dysfunction (*t* = 2.724, *p* = 0.021) showed that publication bias was found. Therefore, the ‘trim and fill” method was performed. Six studies were added and the recalculated prevalence of smell dysfunction according to the “trim and fill” method was 4.46% (95% CI: 0.01–12.74%). For smell or taste dysfunction, publication bias was observed in this study, with the Egger's test being 3.690 (*p* = 0.006). Therefore, the “trim and fill” method was performed. Six studies were added and the recalculated prevalence of smell or taste dysfunction according to the ‘trim and fill' method was 1.21% (95% CI: 0.01–5.50%).

Also, the details of sensitivity analysis are presented in Supplementary Figures 3, 4. When each study was excluded one-by-one, the recalculated combined results did not change significantly. The pooled prevalence of smell dysfunction ranged from 10.35% (95% CI: 5.75–14.95%) to 17.62% (95% CI: 8.57–26.67%), and the I^2^ statistic varied from 81.70 to 94.90%, no individual study significantly influenced the overall results. The pooled prevalence of smell or taste dysfunction ranged from 9.89% (95% CI: 5.56–14.22%) to 18.08% (95% CI: 9.74–26.42%), and the *I*^2^ statistic varied from 96.10 to 98.20%, no individual study significantly influenced the overall results.

## Discussion

### Key Findings

This review has highlighted the importance of considering the smell and/or taste dysfunctions of children with COVID-19. A total of 18 studies were included, four different types of prevalence were reported. The results showed that the pooled prevalence of smell dysfunction among children with COVID-19 was 15.97% (95% CI: 8.18–23.77%), the pooled prevalence of taste dysfunction among children with COVID-19 was 9.20% (95% CI: 4.25–14.16%), the pooled prevalence of smell or taste dysfunction among children with COVID-19 was 15.50% (95% CI: 10.30–20.70%) and the pooled prevalence of smell and taste dysfunction among children with COVID-19 was 20.21% (95% CI: 14.14–26.28%). In the subgroup analyses, several variables including gender, age, the percentage of patients with comorbidities, the percentage of mild or moderate patients, area and quality score were found as significant sources of heterogeneity for the prevalence.

### Comparison With the Literature

The pooled prevalence of smell dysfunction among children with COVID-19 in this study was 15.97% (95% CI: 8.18–23.77%), which was lower than the prevalence among adult patients with COVID-19 (43.00%) (12). In addition, the pooled prevalence of taste dysfunction among children with COVID-19 in this study was 9.20% (95% CI: 4.25–14.16%), which was lower than the prevalence among adult patients with COVID-19 (44.60%) (12). Currently, the pathological mechanisms are still unclear (12). It has been hypothesized that such age differences in prevalence of smell or taste dysfunctions may be caused by age-dependent differences in ACE2 expression (41). The distribution and expression of ACE2 in the oral cavity and in nasal epithelium differ between children and adults, which could contribute to differences in sensory impairment (41). Although the prevalence of smell or taste dysfunctions among children with COVID-19 is lower than that of adults, the prevalence of smell and taste dysfunctions are still relatively high in children with COVID-19. Assessment of initial pathognomonic symptoms among high-risk population for early detection (such as olfactory or gustatory dysfunction) is essential to help preventing the spreading of the disease.

It is said that males were more likely to experience smell loss than females (6). In our study, however, a higher percentage of male participants was associated with lower prevalence of smell dysfunction or taste dysfunction. Also, von Bartheld et al.'s study showed the same results in adults with COVID-19 (12). Future studies should pay more attention to explore the influence of gender on smell and taste dysfunctions among children with COVID-19. Significant difference in the prevalence of smell or taste dysfunction with age was also observed, younger age was associated with lower prevalence of smell or taste dysfunction. Currently, little is known about olfactory or gustatory function in child development, at least in part because there are special challenges in the testing of olfactory or gustatory function in children (42, 43). Due to lack of suitable clinical tests, the measurement of smell and taste function in children is neglected across the world (42). Previous studies indicated that olfactory or gustatory function is typically only tested in children of 4–5 years of age or older (42, 43). This study included children aged younger than 4 years older, so we think the results of this subgroup need to be treated with caution. It is important to explore new methods to test children's sense of smell or taste function that is less dependent on cognitive factors; this may enhance our understanding of young children's olfactory and gustatory capabilities. Previous studies have shown that a smaller sample size generally leads to a higher effect size (44); significant moderation of the prevalence of smell or taste dysfunction by sample size was noted in the current study too, which was consistent with previous results.

Also, previous studies showed that comorbidities can adversely affect the sense of smell or taste, and the effect could be short-term or permanent, this research has yielded consistent results (6, 45). The results of our subgroup analysis indicated that higher percentage of patients with comorbidities was associated with higher prevalence of smell dysfunction or taste dysfunction. Additionally, we tried to explore the impact of the disease course of COVID-19 on smell and taste dysfunctions in the subgroup analysis. The results showed that higher percentage of children with mild or moderate COVID-19 were associated with lower prevalence of smell or taste dysfunction, which differed from studies among adult COVID-19 patients where patients with severe COVID were less likely to develop smell or taste dysfunction (12). Future research is needed to further explore the impact of the disease course on smell and taste dysfunctions in children with COVID-19, and to try to explain the pathological mechanisms.

Vincent et al. (9) found that higher rates of smell dysfunction were reported using expanded tests, compared to brief tests. Since most of the included studies did not report measurement tools on smell or taste dysfunction, we were unable to explore the impact of measurement tools on the pooled prevalence. To fully understand the difference in the prevalence of smell of taste dysfunctions among children with COVID-19 using different assessment tools, the current results require further clarification. Previous studies have shown that higher quality studies are more likely to report a lower prevalence rate (46), but no significant difference was found in this study. It might be related to issues with numbers of studies, no included studies were rated as high quality, and the results may not be entirely representative. Hence, this observation requires further clarification.

### Implications

Epidemiological studies have demonstrated a rather high prevalence of smell or taste dysfunctions among COVID-19 patients (ranged from 43.00 to 47.40%) (12). COVID-19 patients report many clinical symptoms, and most of these symptoms will fade out after the epidemic, but it is not yet clear how many of the COVID-19 related smell or taste dysfunctions are transient or permanent. Some patients reported that they still have symptoms even after 30 days (47). In terms of applicability to COVID-19, evidence from this study suggests that smell or taste dysfunctions were common among children with COVID-19, healthcare policies need to take into account both short-term and long-term preventive strategy of smell or taste dysfunctions in the forthcoming months. Additionally, the clinic should pay more attention to females and patients with comorbidities. During our process of screening data, we found that most of related data were about adults. There were relatively few data about children, only 18 studies were included in the current review. In addition, the quality of most included studies is not very high. Due to lack of data, we were unable to analyze some potentially confounding factors (such as measurement tools, smoking) (48). For the confounding factors we have analyzed, many included studies did not report relevant data, such as average age of the patients, the severity of the disease. Therefore, there might be a considerable amount of uncertainty regarding the pooled prevalence of smell or taste dysfunctions between different subgroups. Regarding the pooled prevalence of smell or taste dysfunction, publication bias was found, so the pooled prevalence was recombined using the “trim and fill” method. Since the sample size varies greatly between different articles (ranged from 35 to 58,963), and an extreme outlier study (with a prevalence <1%) was included, the results of ‘trim and fill' method should be treated with caution too. In this regard, we believe that more reporting of pediatric data in combination with high powered and multi-national studies are needed, to improve our knowledge among children with COVID-19.

Unlike hearing or vision tests, the tests to measure smell and taste loss are not based upon standard, internationally-accepted procedures. Instead, the literature is full of several alternative methods for measuring smell and taste loss (6). Given the impact of measurement tools on the prevalence of smell or taste dysfunctions, more accurate measurement tools or methodologies may have to be developed for children with COVID-19. We think a large multicenter prospective study using a single validated measure of smell or taste dysfunctions and measuring possible confounding factors in randomly selected participants is needed in the future, which would provide a more accurate estimate of smell or taste dysfunctions among children with COVID-19.

### Limitations

First, we excluded studies that were not written in English or Chinese and most included studies were of low or moderate quality. Second, although subgroup analyses were conducted to control many moderating factors for the pooled prevalence of smell or taste dysfunction, heterogeneity remained in this review. It is reported that heterogeneity is difficult to avoid in meta-analysis of epidemiological surveys (9), which suggest the need for caution when drawing inferences about estimates of smell or taste dysfunctions in post-disaster research. Also, the follow-up time varies greatly among the included longitudinal studies, which hinders comparability, we were unable to pool the prevalence. Moreover, few studies used objective assessment methods for establishing the presence of smell or taste dysfunctions, whereas most relied on self-reports. These may lead to bias in the ascertainment of smell or taste dysfunctions. For example, it is possible for patients to confuse taste function and aroma sense perception (49).

## Conclusion

The results showed that the pooled prevalence of smell dysfunction among children with COVID-19 was 15.97% (95% CI: 8.18–23.77%), and the pooled prevalence of taste dysfunction among children with COVID-19 was 9.20% (95% CI: 4.25–14.16%). Higher smell or taste dysfunction rates were associated with being female, younger age, smaller sample size, patients in Asia, and with comorbidities. Further research is needed to identify effective strategies for preventing and treating smell and taste dysfunctions among children with COVID-19.

## Data Availability Statement

The original contributions presented in the study are included in the article/Supplementary Material, further inquiries can be directed to the corresponding author/s.

## Author Contributions

QY, DQ, and XL contributed to the design of the study. DQ and QY screened the text, extracted, and analyzed the data. XG and YH conducted the quality assessment. QY wrote the first draft of the manuscript with input from DQ. All authors approved the final manuscript.

## Conflict of Interest

The authors declare that the research was conducted in the absence of any commercial or financial relationships that could be construed as a potential conflict of interest.

## Publisher's Note

All claims expressed in this article are solely those of the authors and do not necessarily represent those of their affiliated organizations, or those of the publisher, the editors and the reviewers. Any product that may be evaluated in this article, or claim that may be made by its manufacturer, is not guaranteed or endorsed by the publisher.

## Abbreviations

COVID-19: Coronavirus disease 2019
SARS: Severe acute respiratory syndrome.
