# Supplementary material for: Prevalence of Smell or Taste Dysfunction Among Children With COVID-19 Infection: A Systematic Review and Meta-Analysis
Source: Front Pediatr. 2021 Aug 3;9:686600. doi: 10.3389/fped.2021.686600 (PMC8369032; doi:10.3389/fped.2021.686600)

Table S1 PRISMA checklist

Section / topic	#	Checklist item	Reported on page #
**TITLE**			
Title	1	Prevalence of smell or taste dysfunction among children with COVID-19 infection: a systematic review and meta-analysis	Title
**ABSTRACT**			
Structured summary	2	Background: As key symptoms of COVID-19 infection, the prevalence of smell or taste dysfunctions among children with COVID-19 varies greatly across studies. This review aimed at examining the pooled prevalence of smell or taste dysfunctions among children with COVID-19, summarizing possible causes of the inconsistencies in the current estimates.<br><br>Methods: This study is a rapid review and meta-analysis. Systematic searches of databases were conducted for literature published until 12 January 2021. Statistical analyses were performed using R software, the pooled prevalence was combined using random effects model. The Loney criteria was used for quality assessment.<br><br>Results: A total of 18 eligible studies were included, The results showed that the pooled prevalence of smell dysfunction among children with COVID-19 was 15.97% (95% CI: 8.18% to 23.77%), the pooled prevalence of taste dysfunction among children with COVID-19 was 9.20% (95% CI: 4.25% to 14.16%), the pooled prevalence of smell or taste dysfunction among children with COVID-19 was 15.50% (95% CI: 10.30% to 20.70%) and the pooled prevalence of smell dysfunction among children with COVID-19 was 20.21% (95% CI: 14.14% to 26.28%). Higher smell or taste dysfunction rates were associated with being female, younger age, smaller sample size, were patients in Asia and with comorbidities.<br><br>Conclusions: Evidence suggests that smell or taste dysfunctions were common among children with COVID-19. Further research is needed to identify effective strategies for preventing and treating smell and taste dysfunctions among children with COVID-19.	Abstract
**INTRODUCTION**			

Rationale	3	Chemosensory functions, which are usually known as taste and smell functions, are the major pathways for mammals to sense and respond to chemical compounds in the environment, such as odor, flavor, and stimulant. COVID-19-related smell or taste dysfunctions has been described as sudden onset, and may occur in the presence or absence of other key symptoms. Due to increasing awareness of smell or taste dysfunction as potential early symptoms of COVID-19 infection, 'new loss of taste or smell' was added to its list of symptoms that may appear 2 to 14 days after exposure to COVID-19. Understanding the prevalence of smell or taste dysfunction among COVID-19 patients is quite important, which may lead to increases in clinic visits and smell or taste testing due to concerns for COVID-19.	Introduction
Objectives	4	This review aimed at examining the pooled prevalence of smell and/or taste dysfunctions among COVID-19 patients aged < 18, summarizing possible causes of the inconsistencies (such as age, gender, sample size, health status) in the current estimates, try to provide a reference for COVID-19 and possible outbreak of similar infectious diseases in the future.	Introduction
**METHODS**			
Protocol and registration	5	This review was reported in accordance with the PRISMA guideline and MOOSE guidelines.	Methods
Eligibility criteria	6	Studies were included if they meet the following criteria: (1) the study was observational study; (2) information about prevalence of smell or taste dysfunction among children with COVID-19 was provided; (3) the full article was written in English or Chinese; (4) the participants were aged < 18 years old. Studies were excluded: (1) if the report was a review, meta-analysis or protocol; (2) if the study was case series with only selected cases having smell or taste dysfunction were reported.	Methods
Information sources	7	Electronic searches with PubMed, EMBASE, Web of Science, the Cochrane Library, Chinese National Knowledge Infrastructure (CNKI) and PsycArticle were independently conducted by two reviewers. The following search terms were used: 'smell dysfunction' (including smell loss, smell disorder, olfactory dysfunction, etc.); 'taste dysfunction' (including taste loss, taste disorder, gustatory dysfunction, etc.); 'COVID-19' (including COVID-19, SARS-CoV-2, Coronavirus disease 2019 et al.); children (including child, newborns, teenager, adolescent, youngster, etc.). See supplementary data for a full search strategy. Restrictions on the publication date were set, only studies published between 1 December 2019 to 30 October 2020 were searched for. An update search was conducted on 12 January 2021. See supplementary materials for the details. Given that this field is developing rapidly, the preprint servers medRxiv for studies published between Jan 1, 2020, and	Methods

		October 30, 2020, with the term “coronavirus” or “COVID-19” in the title or abstract were also searched for.	
Search	8	**A. Smell or taste dysfunction**<br>1. loss of taste [Title/Abstract]<br>2. loss of smell [Title/Abstract]<br>3. taste dysfunction [Title/Abstract]<br>4. smell dysfunction [Title/Abstract]<br>5. taste loss [Title/Abstract]<br>6. smell loss [Title/Abstract]<br>7. taste [Title/Abstract<br>8. smell sensation [Title/Abstract]<br>9. taste [Title/Abstract]<br>10. smell impairment [Title/Abstract]<br>11. senses of taste [Title/Abstract]<br>12. smell [Title/Abstract]<br>13. smell disorder [Title/Abstract]<br>14. taste disorder [Title/Abstract]<br>15. anosmia [Title/Abstract]<br>16. ageusia [Title/Abstract]<br>17. olfactory dysfunction [Title/Abstract]<br>18. taste alterations [Title/Abstract]<br>19. smell alterations [Title/Abstract]<br>20. taste perception [Title/Abstract]<br>21. smell perception [Title/Abstract]<br>22. smell [Title/Abstract]<br>23. taste problems [Title/Abstract]	Supplementary data

		24. clinical characteristics [Title/Abstract]<br>25. 1 OR 2 OR 3 OR 4 OR 5 OR 6 OR 7 OR 8 OR 9 OR 10 OR 11 OR 12 OR 13 OR 14 OR 15 OR 16 OR 17 OR 18 OR 19 OR 20<br>**B. COVID-19**<br>26. COVID-19 [Title/Abstract]<br>27. Coronavirus disease 2019 [Title/Abstract]<br>28. Covid 19 [Title/Abstract]<br>29. severe acute respiratory syndrome coronavirus 2[Title/Abstract]<br>30. SARS-CoV-2 [Title/Abstract]<br>31. SARS-CoV [Title/Abstract]<br>32. novel coronavirus [Title/Abstract]<br>33. coronavirus [Title/Abstract]<br>34. CoV-2 [Title/Abstract]<br>35. 2019-nCoV [Title/Abstract]<br>36. SARS COV2 [Title/Abstract]<br>37. 21 OR 22 OR 23 OR 24 OR 25 OR 26 OR 27 OR 28 OR 29 OR 30 OR 31<br>**C. Children**<br>38. Child<br>39. Children<br>40. Adolescent<br>41. Newborns<br>42. Teenager<br>43. Youngster<br>44. Young<br>45. 38 OR 39 OR 40 OR 41 OR 42 OR 43 OR 44 OR 45<br>46. 25 AND 37 AND 46	

Study selection	9	Studies were included if they meet the following criteria: (1) the study was observational study; (2) information about prevalence of smell or taste dysfunction among children with COVID-19 was provided; (3) the full article was written in English or Chinese; (4) the participants were aged < 18 years old. Studies were excluded: (1) if the report was a review, meta-analysis or protocol; (2) if the study was case series with only selected cases having smell or taste dysfunction were reported.	Methods
Data collection process	10	Two reviewers (DQ and QZY) checked the titles, abstracts and full-texts of the initial search results independently. Any discrepancies that emerged in these procedures were discussed and resolved by involving a third reviewer (XL).	Methods
Data items	11	Two reviewers (DQ and QZY) checked the titles, abstracts and full-texts of the initial search results independently. Data were extracted on first author, year of publication, country or area, sample size, response rate, percentage of male participants, average age of participants, instruments used to identify smell or taste dysfunction, prevalence of smell and/or taste dysfunctions, percentage of mild or moderate patients, percentage of patients with comorbidity, quality score of the included studies, etc.	Methods
Risk of bias in individual studies	12	Two independent reviewers (XLG and YXH) used the established guidelines, the Loney criteria, to evaluate the methodological quality of the included studies, which has been widely used to evaluate observational studies. The included papers were scored according to eight criteria, such as study design, definition of participants, response rate, sampling method, sample size, appropriateness of measurement and analysis. The scores range from 0 to 8, with a score of 0-3 as low quality, 4-6 as moderate and 7-8 as high	Methods
Summary measures	13	prevalence of smell and/or taste dysfunctions	Methods
Synthesis of results	14	When data were available for three or more studies, prevalence was combined. When there were four or more studies, quantitative subgroup analysis, analysis of publication bias and sensitivity analysis was conducted. All the statistical analyses were performed using the "meta" (4.12-0) and "metafor" package (2.4-0) of R version 4.0.2. Between-study heterogeneity was evaluated by Cochran's Q test and quantified by the I^2 statistic, with values 50% or more indicating possible heterogeneity. The pooled prevalence of smell and/or taste dysfunctions was combined using random effects model if significant heterogeneity was observed across studies (when $P < 0.05$, $I^2 > 50\%$).	Methods

Section/topic	#	Checklist item	Reported on page #
Risk of bias across studies	15	Publication bias was investigated by funnel plot and Egger's test. To evaluate the consistency of the results, sensitivity analysis was performed by removing each study individually. All the statistical tests were 2-sided, with a significance threshold of $P < 0.05$.	Methods
Additional analyses	16	In order to compare the prevalence from different studies (such as age, gender, sample size, health status, etc.), we conducted quantitative subgroup analysis. The difference between subgroups was examined using the Cochran's Q chi-square tests.	Methods
**RESULTS**			
Study selection	17	From the initially identified 2377 records, 1392 records remain after duplicates were excluded. 1135 references were excluded on the basis of the title or abstract, leaving 257 full-text studies for further scrutiny. Of these, 18 met the selection criteria. There were 239 studies were excluded due to the following reasons: no data on prevalence of smell and/or taste dysfunction (n=157); duplicate publications (n=4); no full-text (n=9); review (n=3); not for COVID-19 patients < 18 (n=59); included non-COVID-19 patients (n=7). Finally, 18 articles were included for analysis. See Fig.1 for the details.	Results
Study characteristics	18	As presented in Table 1, 18 articles met the inclusion criteria. Of the included studies, fifteen were cross sectional studies, two were longitudinal designs and one were case control studies. Most of the included studies were from Asia, such as China, south Korea, and Turkey. See Table 1 for the details. From the 18 papers, no study was rated as high quality, sixteen (88.88%) were rated as moderate, and two (11.12%) were rated as low quality. Details of the methodological quality assessments of all 18 studies are showed in Table S3 and Table S4.	Results
Risk of bias within studies	19	From the 18 papers, no study was rated as high quality, sixteen (88.88%) were rated as moderate, and two (11.12%) were rated as low quality. Details of the methodological quality assessments of all 18 studies are showed in Table S3 and Table S4.	Results
Results of individual studies	20	The forest plots in Fig. 2 depict the details. A total of 18 studies reported prevalence of smell and/or taste dysfunction, most of them reported more than one type of prevalence among COVID-19 patients. Specifically, 11 of the included studies reported the prevalence on smell dysfunction [12, 24-32], 9 of the included studies reported prevalence on taste dysfunction	Results

		[12, 25, 26, 29, 32-35], 10 of the included studies reported prevalence on smell or taste dysfunction [12, 13, 24, 26, 31, 36-40], and 2 of the included studies reported prevalence on smell and taste dysfunction [12, 26]. Thus, four different types of prevalence were combined.	
Synthesis of results	21	The prevalence of smell dysfunction reported among the included studies ranged from 1.23% to 68.35%. A total of 779 children with COVID-19 were identified in the 12 articles, of which 135 were reported with smell dysfunction. The random effects model was used to determine the pooled prevalence (I2 = 94.40%, P < 0.001), the pooled prevalence of smell dysfunction among children with COVID-19 was 15.97%, with a 95% CI of 8.18% to 23.77%. See Fig. 2 for the details.<br>The prevalence of taste dysfunction reported among the included studies ranged from 0.00% to 24.11%. A total of 593 children with COVID-19 were identified in the 9 articles, of which 70 were reported with taste dysfunction. The random effects model was used to determine the pooled prevalence (I2 = 82.00%, P < 0.001), the pooled prevalence of taste dysfunction among children with COVID-19 was 9.20%, with a 95% CI of 4.25% to 14.16%. See Fig. 2 for the details.<br>The prevalence of smell or taste dysfunction reported among the included studies ranged from 0.45% to 72.22%. A total of 61748 children with COVID-19 were identified in the 10 articles, of which 574 were reported with smell or taste dysfunction. The random effects model was used to determine the pooled prevalence (I2 = 98.00%, P < 0.001), the pooled prevalence of smell or taste dysfunction among children with COVID-19 was 15.50%, with a 95% CI of 10.30% to 20.70%. See Fig. 2 for the details.<br>The prevalence of smell and taste dysfunction reported among the included studies ranged from 19.86% to 22.22%. A total of 168 children with COVID-19 were identified in the 2 articles, of which 34 were reported with both smell and taste dysfunction. The fix effects model was used to determine the pooled prevalence (I2 = 0.00%, P < 0.001), the pooled prevalence of smell and taste dysfunction among children with COVID-19 was 20.21%, with a 95% CI of 14.14% to 26.28%. See Fig. 2 for the details.	Results
Risk of bias across studies	22	Funnel plots for different outcomes (smell dysfunction, taste dysfunction and smell or taste dysfunction) were presented in Fig.S1 and Fig.S2. The results of visual funnel plot and the Egger's test for smell dysfunction (t =2.724, p = 0.021) and showed that publication bias was not found. Therefore, the 'trim and fill' method was performed. Six studies were added and the recalculated prevalence of smell dysfunction according to the 'trim and fill' method was 4.46% (95% CI: 0.01–12.74%). For smell or taste dysfunction, publication bias was observed in this study, for the Egger's test being 3.690 (p = 0.006).	Results

		Therefore, the 'trim and fill' method was performed. Six studies were added and the recalculated prevalence of smell or taste dysfunction according to the 'trim and fill' method was 1.21% (95% CI: 0.01–5.50%).<br>Also, the details of sensitivity analysis are presented in Fig. S3 and Fig. S4. When each study was excluded one-by-one, the recalculated combined results did not change significantly. The pooled prevalence of smell dysfunction ranged from 10.35% (95% CI: 5.75-14.95%) to 17.62% (95% CI: 8.57-26.67%), and the I2 statistic varied from 81.70% to 94.90%, no individual study significantly influenced the overall results. The pooled prevalence of smell or taste dysfunction ranged from 9.89% (95% CI: 5.56-14.22%) to 18.08% (95% CI: 9.74-26.42%), and the I2 statistic varied from 96.10% to 98.20%, no individual study significantly influenced the overall results.	
Additional analysis	23	The details of subgroup analyses, publication bias and sensitivity analysis are presented in Table 2, Table 3, Fig. S1, Fig. S2, Fig. S3, Fig. S4.	Results
**DISCUSSION**			
Summary of evidence	24	This review has highlighted the importance of considering the smell and/or taste dysfunctions of children with COVID-19. A total of 18 studies were included, four different types of prevalence were reported. The results showed that the pooled prevalence of smell dysfunction among children with COVID-19 was 15.97% (95% CI: 8.18% to 23.77%), the pooled prevalence of taste dysfunction among children with COVID-19 was 9.20% (95% CI: 4.25% to 14.16%), the pooled prevalence of smell or taste dysfunction among children with COVID-19 was 15.50% (95% CI: 10.30% to 20.70%) and the pooled prevalence of smell dysfunction among children with COVID-19 was 20.21% (95% CI: 14.14% to 26.28%). In the subgroup analyses, several variables including gender, age, the percentage of patients with comorbidities, the percentage of mild or moderate patients, area and quality score were found as significant sources of heterogeneity for the prevalence.	Discussion
Limitations	25	First, we excluded studies were not written in English or Chinese and most included studies were of low or moderate quality. Second, although subgroup analyses were conducted to control many moderating factors for the pooled prevalence of smell or taste dysfunction, heterogeneity was still remained in this review. It is reported that heterogeneity is difficult to avoid in meta-analysis of epidemiological surveys [9], which suggesting the need for caution when drawing inferences about estimates of smell or taste dysfunctions in post-disaster research. Also, the follow-up time varies greatly among the included longitudinal studies, which hinders comparability, we were unable to pool the prevalence. Moreover, few studies used objective assessment methods for establishing the presence of smell or taste dysfunctions, whereas most relied on self-	Discussion

		reports. These may lead to bias in the ascertainment of smell or taste dysfunctions. For example, it is possible for patients to confuse taste function and aroma sense perception.	
Conclusions	26	Evidence suggests that the symptoms of PTSD were very common among people exposed to the trauma resulting from infectious diseases outbreak and may last for a prolonged time. Healthcare policies need to take into account both short-term and long-term preventive strategy of PTSD in the forthcoming months.	Discussion
**FUNDING**			
Funding	27	This research was supported by the Health Commission of Hunan Province (Grant NO: B2017167) and Hunan Pharmaceutical Association (Grant NO: Hn2017007).	Funding

From: Moher D, Liberati A, Tetzlaff J, Altman DG, The PRISMA Group (2009). Preferred Reporting Items for Systematic Reviews and Meta-Analyses: The PRISMA Statement. PLoS Med 6(6): e1000097. doi:10.1371/journal.pmed1000097

For more information, visit: **www.prisma-statement.org**.

Page 2 of 2

Table S2 **MOOSE Checklist**

**Criteria**		**Brief description of how the criteria were handled in the meta-analysis**
**Reporting of background should include**		
√	Problem definition	Smell and taste dysfunctions are common and has been reported as an early indicator of COVID-19, while the prevalence of smell and taste dysfunctions among children with COVID-19 varies greatly across studies, which remains to be summarized quantitatively.
√	Hypothesis statement	smell or taste dysfunctions were very common among children with COVID-19, relevant study characteristics, such as smoking, outcome measures, gender have an impact on the outcome.
√	Description of study outcomes	prevalence of smell and/or taste dysfunctions

√	Type of exposure or intervention used	COVID-19 infection
√	Type of study designs used	We included case-control studies, prospective cohort studies, cross-sectional studies
√	Study population	Children with COVID-19
**Reporting of search strategy should include**		
√	Qualifications of searchers	The credentials of the two investigators DQ and QZY are indicated in the author list.
√	Search strategy, including time period included in the synthesis and keywords	The following search terms were used: 'smell dysfunction' (including smell loss, smell disorder, olfactory dysfunction, etc.); 'taste dysfunction' (including taste loss, taste disorder, gustatory dysfunction, etc.); 'COVID-19' (including COVID-19, SARS-CoV-2, Coronavirus disease 2019 et al.); children (including child, newborns, teenager, adolescent, youngster, etc.). See supplementary data for a full search strategy. Restrictions on the publication date were set, only studies published between 1 December 2019 to 30 October 2020 were searched for. An update search was conducted on 12 January 2021. See supplementary materials for the details.
√	Databases and registries searched	Electronic searches with PubMed, EMBASE, Web of Science, the Cochrane Library, Chinese National Knowledge Infrastructure (CNKI) and PsycArticle were independently conducted by two reviewers.<br>The preprint servers medRxiv for studies published between Jan 1, 2020, and October 30, 2020, with the term "coronavirus" or "COVID-19" in the title or abstract were also searched for.
√	Search software used, name and version, including special features	We did not employ a search software. EndNote was used to merge retrieved citations and eliminate duplications
√	Use of hand searching	We hand-searched bibliographies of retrieved papers for additional references
√	List of citations located and those excluded, including justifications	Details of the literature search process are outlined in the flow chart and supplementary data. The citation list is available upon request
√	Method of addressing articles	We excluded studies not in English and Chinese

	published in languages other than English	
√	Method of handling abstracts and unpublished studies	We planned to contacted authors for unpublished studies during the screening process when necessary, no such abstracts and unpublished studies appears in articles that meet the inclusion criteria at last.
√	Description of any contact with authors	Not applicable (All articles that meet the inclusion criteria have complete data for pooled prevalence)
**Reporting of methods should include**		
√	Description of relevance or appropriateness of studies assembled for assessing the hypothesis to be tested	Detailed inclusion and exclusion criteria were described in the methods section.
√	Rationale for the selection and coding of data	Two reviewers (DQ and QZY) checked the titles, abstracts and full-texts of the initial search results independently. Data were extracted on first author, year of publication, country or area, survey period, sample size, response rate, percentage of male participants, average age of participants, instruments used to identify smell or taste dysfunction, prevalence of smell and/or taste dysfunctions, quality score of the included studies, etc. Any discrepancies that emerged in these procedures were discussed and resolved by involving a third reviewer (XL).
√	Assessment of confounding	In order to compare the prevalence from different studies (such as gender, age, diagnostic method etc.), we conducted subgroup meta-analysis. The difference between subgroups was examined using the Cochran's Q chi-square tests.
√	Assessment of study quality, including blinding of quality assessors; stratification or regression on possible predictors of study results	Two independent reviewers (XLG and YXH) used the established guidelines, the Loney criteria, to evaluate the methodological quality of the included studies, which has been widely used to evaluate observational studies. The included papers were scored according to eight criteria, such as definition of participants, study design, sampling method, response rate, sample size, appropriateness of measurement and analysis. The scores range from 0 to 8, with a score of 0-3 as low quality, 4-6 as moderate and 7-8 as high. See Table S3

		for details on the quality assessment.<br>Publication bias was investigated by funnel plot and Egger's test.
√	Assessment of heterogeneity	Heterogeneity of the studies were explored within two types of study designs using Cochrane’s Q test of heterogeneity and I^2 statistic that provides the relative amount of variance of the summary effect due to the between-study heterogeneity.
√	Description of statistical methods in sufficient detail to be replicated	Description of methods of meta-analyses, sensitivity analyses and assessment of publication bias are detailed in the methods.
√	Provision of appropriate tables and graphics	We included 1 flow chart,1 summary table, 1 forest plots for 4 different outcome, 2 tables of subgroup analyses.<br>In addition, we included 4 supplementary Figs (2 funnel plots of publication bias and 2 funnel plots of sensitivity analysis) and 3 supplementary tables in the supplementary data file.
**Reporting of results should include**		
√	Graph summarizing individual study estimates and overall estimate	Figure 2
√	Table giving descriptive information for each study included	Table 1
√	Results of sensitivity testing	Fig S3, Fig. S4
√	Indication of statistical uncertainty of findings	95% confidence intervals were presented with all summary estimates, I^2 values and results of sensitivity analyses
**Reporting of discussion should include**		

√	Quantitative assessment of bias	The results of visual funnel plot and the Egger's test showed that publication bias was found. Therefore, the 'trim and fill' method was performed. The results of visual funnel plot and the Egger's test for smell dysfunction (t =2.724, p = 0.021) and showed that publication bias was not found. Therefore, the 'trim and fill' method was performed. Six studies were added and the recalculated prevalence of smell dysfunction according to the 'trim and fill' method was 4.46% (95% CI: 0.01–12.74%). For smell or taste dysfunction, publication bias was observed in this study, for the Egger's test being 3.690 (p = 0.006). Therefore, the 'trim and fill' method was performed. Six studies were added and the recalculated prevalence of smell or taste dysfunction according to the 'trim and fill' method was 1.21% (95% CI: 0.01–5.50%). Sensitivity analysis showed that no individual study significantly influenced the overall results. However, the observed heterogeneity should be noticed.
√	Justification for exclusion	We excluded studies that not write in English or Chinese, which was a limitation in this review.
√	Assessment of quality of included studies	We discussed the results of the subgroup analyses, and potential reasons for the observed heterogeneity.
**Reporting of conclusions should include**		
√	Consideration of alternative explanations for observed results	We noted that the variations in the prevalence may be due to gender, age or to percentage of mild or moderate patients etc.
√	Generalization of the conclusions	Evidence suggests that the symptoms of smell and/or taste dysfunction were common among children with COVID-19. Healthcare policies need to take into account both short-term and long-term preventive strategy of smell or taste dysfunction in the forthcoming months.
√	Guidelines for future research	At first, healthcare policies need to take into account both short-term and long-term preventive strategy of smell or taste dysfunction in the forthcoming months. Second, children with COVID-19 reported many clinical symptoms, and most of these symptoms will fade out after the epidemic, but it is not yet clear whether the COVID-19 related smell or taste dysfunctions are transient or permanent. Additionally, the clinic should pay more attention to female and patients with comorbidities. Also, we think a large

		multicenter prospective study using a single validated measure of smell and taste dysfunctions, measuring possible confounding factors in randomly selected participants is needed in the future, which would provide a more accurate estimate of smell or taste dysfunctions among children with COVID-19 patients. At last, we think ongoing surveillance is essential.
√	Disclosure of funding source	This research was supported by the Health Commission of Hunan Province (Grant NO: B2017167) and Hunan Pharmaceutical Association (Grant NO: Hn2017007). The funding agency did not take part in the design of the study and collection, analysis, and interpretation of data and in writing the manuscript.

Search strategy

A. Smell or taste dysfunction

1. loss of taste [Title/Abstract]
2. smell [Title/Abstract]
3. taste dysfunction [Title/Abstract]
4. smell dysfunction [Title/Abstract]
5. taste loss [Title/Abstract]
6. smell loss [Title/Abstract]
7. taste [Title/Abstract
8. smell sensation [Title/Abstract]
9. taste [Title/Abstract]
10. smell impairment [Title/Abstract]
11. senses of taste [Title/Abstract]
12. smell [Title/Abstract]
13. smell disorder [Title/Abstract]
14. taste disorder [Title/Abstract]
15. anosmia [Title/Abstract]

16. ageusia [Title/Abstract]
17. olfactory dysfunction [Title/Abstract]
18. taste alterations [Title/Abstract]
19. smell alterations [Title/Abstract]
20. taste perception [Title/Abstract]
21. smell perception [Title/Abstract]
22. smell [Title/Abstract]
23. taste problems [Title/Abstract]
24. clinical characteristics [Title/Abstract]
25. 1 OR 2 OR 3 OR 4 OR 5 OR 6 OR 7 OR 8 OR 9 OR 10 OR 11 OR 12 OR 13 OR 14 OR 15 OR 16 OR 17 OR 18 OR 19 OR 20

B. COVID-19

26. COVID-19 [Title/Abstract]
27. Coronavirus disease 2019 [Title/Abstract]
28. Covid 19 [Title/Abstract]
29. severe acute respiratory syndrome coronavirus 2[Title/Abstract]
30. SARS-CoV-2 [Title/Abstract]
31. SARS-CoV [Title/Abstract]
32. novel coronavirus [Title/Abstract]
33. coronavirus [Title/Abstract]
34. CoV-2 [Title/Abstract]
35. 2019-nCoV [Title/Abstract]
36. SARS COV2 [Title/Abstract]
37. 21 OR 22 OR 23 OR 24 OR 25 OR 26 OR 27 OR 28 OR 29 OR 30 OR 31

C. Children

38. Child
39. Children
40. Adolescent
41. Newborns
42. Teenager
43. Adolescent
44. Youngster
45. Young
46. 38 OR 39 OR 40 OR 41 OR 42 OR 43 OR 44 OR 45

47\. AND 37 AND 46

Table S3 Items of quality assessment

1	Is the target population clearly defined?
2	Was either of the following ascertainment methods used [must be one or the other]? (1) probability sampling, or (2) entire population surveyed
3	Is the response rate >70%
4	Are non-responders clearly described?
5	Is the sample representative of the target population (>300 subjects)?
6	Were data collection methods standardized?

7	Were validated criteria used to assess for the presence/absence of disease?
8	Are the estimates of prevalence given with confidence intervals and in detail by subgroup (if applicable)?

Table S4 Quality assessment of included studies

study	Item 1	Item 2	Item 3	Item 4	Item 5	Item 6	Item 7	Item 8	Total score
Yury Rusetsky et al.	1	0	1	0	0	1	1	1	5
Chenghao Qiu et al.	1	1	0	0	0	1	1	1	5
Jiahui Li et al.	1	1	0	0	0	1	1	1	5
Rebecca Laws et al.	1	1	0	0	0	0	0	1	3
Lakshit Kumar et al.	1	1	1	0	0	0	0	1	4
Nina Krajcar et al.	1	1	1	0	0	0	0	1	4
Muhammet Furkan Korkmaz et al.	1	1	1	0	0	0	0	1	4
James A. King et al.	1	1	1	0	1	0	0	1	5
Muna M. Kilani et al.	1	1	1	0	0	0	0	1	4
Ming He et al.	1	1	1	0	0	0	0	1	4

Mi Seon Han et al.	1	1	1	0	0	0	0	1	4
Mathew Goss et al.	1	1	1	0	0	0	0	1	4
Louise Gaborieau et al.	1	1	1	0	0	0	1	1	4
Talita Duarte-Salles et al.	1	1	0	0	1	0	0	1	4
Ana Concheiro-Guisan et al.	1	1	0	0	0	0	0	1	3
Gilbert Chua et al.	1	1	1	0	0	0	0	1	4
Gazi Arslan et al.	1	1	1	0	0	0	0	1	4
Idris Adedeji et al.	1	1	1	0	0	0	0	1	4

Table S5 subgroup analysis for smell or taste dysfunction

Subgroup	Number of studies	Pooled prevalence % (95%CI)	I^2 (%)	Test of Difference within Each Subgroup	
				Q	p
**Mean age**				7.39	0.006
0-10	2	55.46 (21.02-89.90)	90.80		
>10	6	7.15 (4.56-10.46)	72.20		
**Percentage of male participants (%)**				2.45	0.117
0-50	3	32.07 (4.57-59.56)	98.70		
>50	5	9.46 (2.62-16.30)	89.20		
**Sample size**				6.40	0.011
<300	8	21.04 (9.96-32.12)	99.30		

≥300	2	4.05 (0.01-11.15)	96.40		
**Percentage of patients with mild or moderate COVID-19 (%)**				5.38	0.020
0-50	1	37.04 (19.82-55.25)	-		
>50	2	12.92 (3.76-22.07)	83.8-		
**Percentage of patients with comorbidities (%)**				0.81	0.369
0-10	1	8.52 (0.06-17.37)	-		
≥10	3	9.00 (4.40-12.65)	86.50		
**Area**				2.78	0.248
Asia	5	22.97 (4.89-41.05)	97.60		
America	3	6.90 (0.01-14.14)	94.60		
Europe	1	7.70 (6.53-8.87)	-		

Fig. S1 Funnel plot of smell dysfunctions

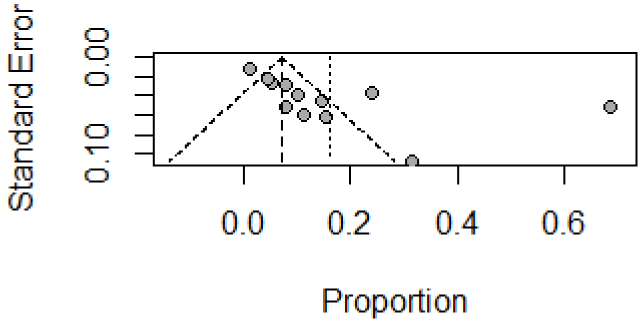

Fig. S2 Funnel plot of smell or taste dysfunctions

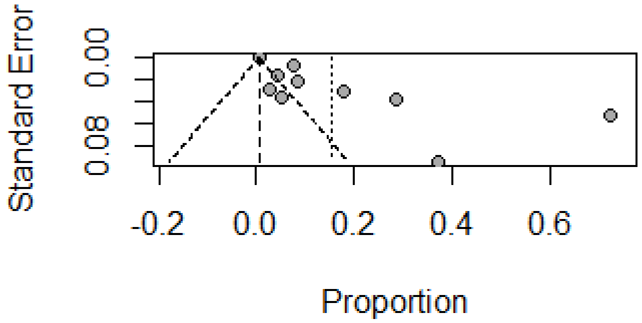

Fig. S3 Results of sensitivity analysis for smell dysfunction

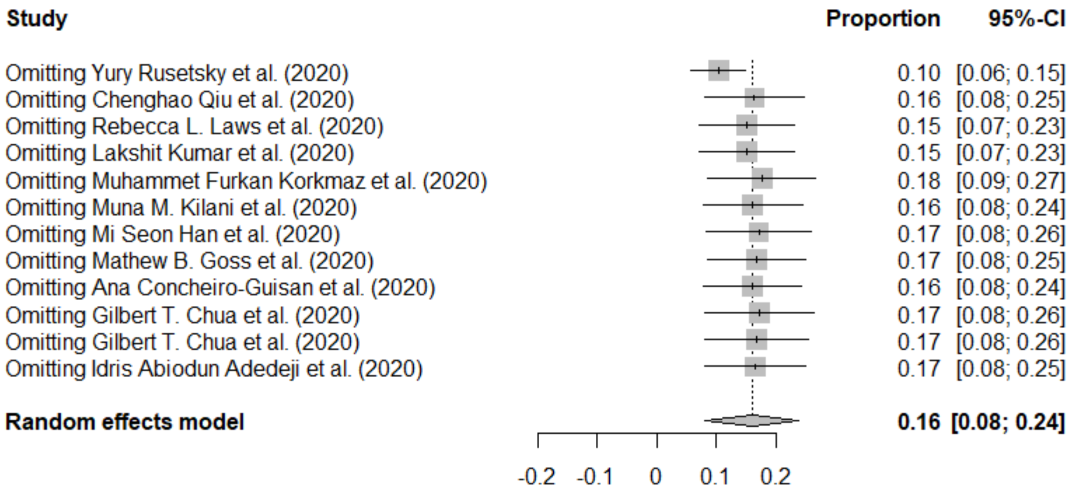

Fig. S4 Results of sensitivity analysis for smell or taste dysfunction

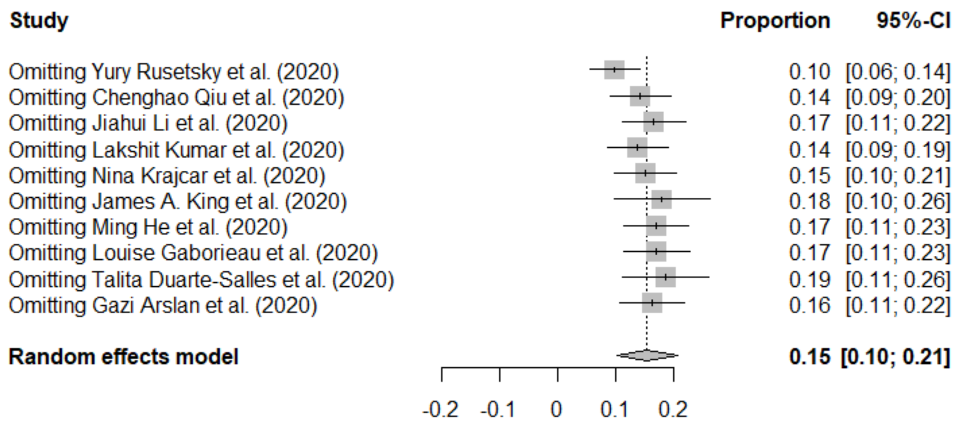

Fig. S5 Forest plot of pooled prevalence of smell or taste dysfunction (without extreme case)

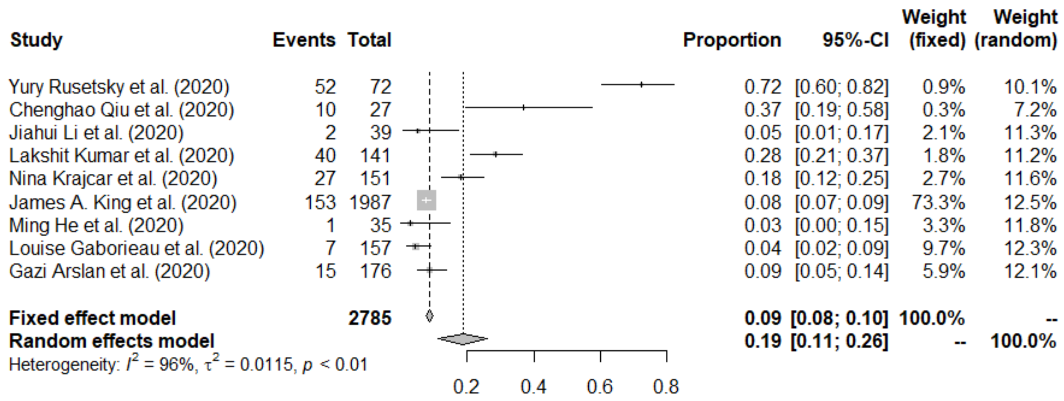

Supplement: Supplementary file 1 [file Data_Sheet_1.PDF]
